# Health-related quality of life in Hodgkin lymphoma: a systematic review

**DOI:** 10.1186/s12955-016-0515-6

**Published:** 2016-07-29

**Authors:** Nadine Linendoll, Tully Saunders, Rebecca Burns, Jonathan D. Nyce, Kristen B. Wendell, Andrew M. Evens, Susan K. Parsons

**Affiliations:** 1 Tufts Cancer Center, Tufts Medical Center, 800 Washington Street, #245, Boston, MA 02111 USA; 2 Institute for Clinical Research and Health Policy, Tufts Medical Center, 800 Washington Street, #345, Boston, MA 02111 USA; 3 Advocate Lutheran General Hospital, 1775 Dempster St, Park Ridge, IL 60068 USA; 4 Tufts University School of Medicine, 145 Harrison Avenue, Boston, MA 02111 USA

**Keywords:** Hodgkin lymphoma, Quality of life, Survivorship, Systematic review

## Abstract

**Purpose:**

Hodgkin Lymphoma (HL) is highly curable with well-established treatment regimens; however, the impact on patient’s health-related quality of life (HRQL) from diagnosis through survivorship is unclear. This systematic review aimed to describe the available literature on HRQL in HL, assess the quality of these studies, identify gaps in the literature and recommend further areas of research.

**Methods:**

Following PRISMA guidelines, we performed a systematic review to include studies assessing the HRQL in HL patients. Articles identified through database searches were screened and data extracted. Quality was evaluated using a 6-point scale, adapted from published HRQL systematic reviews.

**Results:**

Sixty five articles published between 1986 and 2015 met inclusion criteria. These included 53 (82 %) cross-sectional studies; 12 (18 %) longitudinal studies, including three embedded in randomized trials; and three additional longitudinal studies that began assessment at diagnosis. Study sample sizes of HL patients varied considerably with only five (42 %) longitudinal studies including more than 50 patients. Multidimensional HRQL was assessed in 45 studies, single HRQL domains in 22 studies, and symptoms, including fatigue, in 28 studies.

**Conclusions:**

The majority of studies employed a cross-sectional design, enrolling HL survivors at least 10 years after the completion of therapy. Emphasis on HRQL following therapy may inform initial treatment decisions and long-term survivorship goals. We recommend that future research include prospective, longitudinal randomized designs across both treatment and time.

## Background

Hodgkin Lymphoma (HL) has well established treatment regimens that have yielded highly effective, long-term cure rates [1, 2]. In 2015, there were an estimated 9050 new cases of the disease, which is a much lower incidence than more commonly occurring cancers. The number of persons living with HL in 2012, however, was estimated to approach 190,000 [3]. Thus, from an oncologic perspective the successful treatment for HL over the last 25 years has led to a high number of long-term survivors.

The incidence of HL is bimodal by age with the first peak within adolescence and young adulthood, ages 15–40, and the second after age 55 [4]. For younger people with HL, the aggressive cancer treatment often interrupts important developmental milestones, such as graduation from high school or college, establishing relationships, finding a first apartment or getting a job. Despite the decades of curative therapy for HL, comparatively little is known about how HL affects health-related quality of life (HRQL)—through diagnosis and treatment. Therefore, healthcare providers lack the information that they need regarding how best to intervene in a timely and effective manner to mediate HL’s acute and late-term effects.

HRQL is a multidimensional construct, which reflects the World Health Organization’s definition of health as incorporating physical, mental, and social health [5]. The history of HRQL assessment largely parallels the advances in HL therapy with most of the instrument development and large-scale validation studies for adults commencing in the 1980’s. The first randomized controlled trial (RCT) containing an HRQL outcome was reported in 1986 by Croog and Levine [6]. In contrast, instrument development for children lagged considerably, but psychometrically robust measures are now available for most patients across the age continuum. Several validated instruments address the multidimensional construct, while others focus on individual dimensions or domains of HRQL, such as physical or psychosocial functioning. Still others measure individual symptoms, such as pain, nausea or fatigue. The selection of a particular instrument or instruments is informed by the particular study question.

Although treatment regimens are well-established, the impact on HL patient’s HRQL from diagnosis through long-term survivorship is still unclear. In this study, we report on a systematic review that we conducted on the HRQL literature to answer the following research question: What is the HRQL impact of HL and its treatment as reflected in the current literature? Our goals were to: (1) describe the available literature on HRQL in HL, (2) assess the quality of these studies and, (3) identify gaps in the available literature and recommend further areas of research.

## Methods

### Literature search strategy

**Fig. 1:**
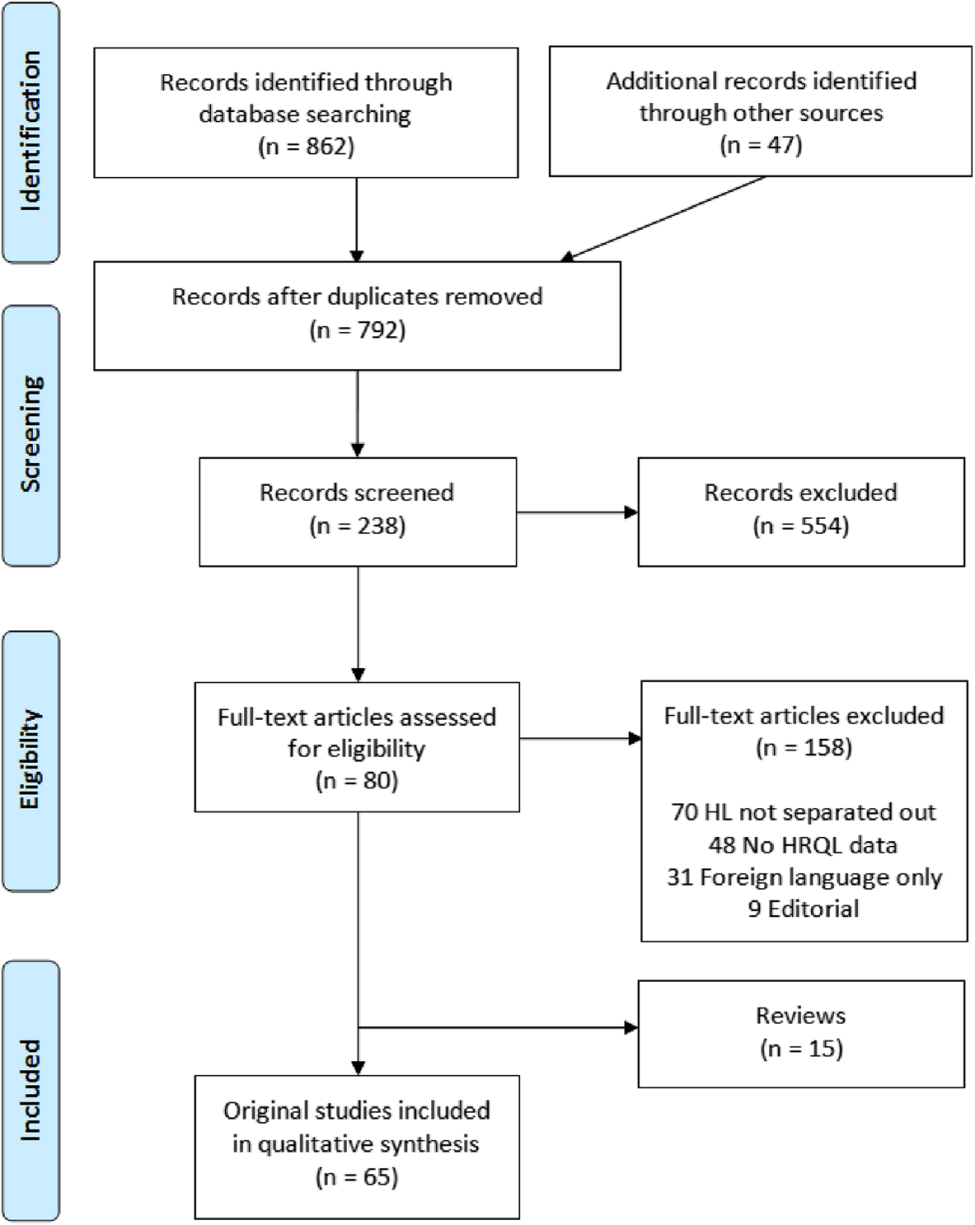
Systematic review flow diagram following PRISMA guidelines

Our review was guided by the PRISMA statement [7]. We searched Medline, CINAHL and PsychInfo using Medical Subject Headings and keywords, such as *Hodgkin disease, quality of life, health- related quality of life, well-being, functional status, health status* and *experiential health status* for articles published since inception to end of May 2016 that reported primary HRQL data specific to HL patients. A priori, we sought studies that assessed single or multi-dimensional HRQL domains or discrete symptoms, such as fatigue. Study design was not part of the inclusion/exclusion criteria as our goal was to examine the entirety of the HRQL in HL literature. Studies were excluded if they (1) did not report primary HRQL separately for HL patients or (2) were not available in English. Following this database search, a citation analysis was performed on review articles to identify additional articles (see Fig. 1). This was done to ensure that this review afforded the most comprehensive representation of the literature. Selected articles included HL patients across the age (children and adults) and care continuum, from initial diagnosis through long-term survivorship.

### Review of the abstracts and full-text articles

The six-member team initially reviewed a training set of 50 abstracts for consideration in the study, applying established inclusion/exclusion criteria to ensure consistency across all team members. Subsequently, teams of two reviewers independently applied inclusion/exclusion criteria to the remaining abstracts and then full text articles, resolving any discrepancies through consensus. Full text articles meeting inclusion criteria were independently data extracted using a standardized data extraction form and checked for accuracy by a second review.

Review articles were handled differently from original reports. Because the review articles did not include original data, we did not perform data extraction on review articles. The review articles provided background information and were used in the citation analysis.

### Quality indicators

The methodological quality of each study was evaluated by a set of six predetermined criteria, adapted from previously published systematic reviews [8, 9]. Quality assessment (QA) criteria included: (1) description of ≥2 demographic variables specific to HL patients, such as age and gender; (2) ≥2 clinical characteristics, specific to HL patients, such as stage or site of disease; (3) sample size ≥50 HL participants; (4) HL-specific HRQL scores; presented as a mean summary score and measure of variability in either table or text format; (5) analysis of HRQL scores by HL specific demographic characteristics and (6) HL results compared within groups. The final QA checklist scores were summed by each article to give an overall quality score, ranging from 0 to 6 in which higher scores indicated higher quality.

## Results

The database search totaled 792 unique records. Following the screening process, 80 studies, published between 1986 and 2015, were identified, including 65 primary studies and 15 review articles. Reasons for rejection of full text articles included HL data not separated out, no HRQL data reported, not available in English, or an editorial article. The following section reports on the 65 primary studies.

### Study characteristics

Study characteristics are presented in Table 1. Of the 65 studies, 53 (82 %) utilized cross-sectional design; only 12 (18 %) used longitudinal design. Of the 12 longitudinal studies, seven included individuals who were off treatment, while five included samples who were both on and off treatment. We identified three longitudinal RCTs [10–12], of which patients were followed from the time of diagnosis through up to 10 years of survivorship. These studies accrued large samples and made comparison of HRQL according to randomized treatment groups. The 53 cross sectional studies reported on 35 unique cohorts and only one included patients both on and off treatment. The remaining 52 (98 %) cross-sectional studies only included individuals who were off active treatment.

**Table 1:** Study characteristics (*N* = 65)

Study design, *n* (%)	
Comparator: RCT	3 (5)
Comparator: cohort	14 (22)
Cohort	45 (69)
Comparator: case control	3 (5)
Study type, *n* (%)	
Longitudinal	12 (18)
Cross-sectional	53 (82)
Study setting, *n* (%)	
In person	18 (28)
Remote	42 (65)
NS	5 (8)
Decade of publication	
Before 1995	6 (9)
1995 to 2004	25 (38)
2005 to June 2015	34 (52)
Study sponsor, *n* (%)	
Cooperative group	7 (11)
Investigator initiated	58 (89)
Geographic location, *n* (%)	
USA	19 (29)
Norway	17 (26)
Netherlands	7 (11)
Germany	4 (6)
Other Europe	10 (15)
South America	4 (6)
Canada	3 (5)
Asia	1 (2)
*n* (HL patients)	
Median (range)	132 (12–3208)
25–75^th^ percentile	58–280
Treatment status, *n* (%)	
Active and off treatment	6 (9)
Off treatment only	59 (91)
Age^a^	
Mean (*n* = 33)	39.6
Median (*n* = 21)	38
Gender, %	
Female (*n* = 54)	44.0 %
Range	0–100 %

^a^Age: 3 studies with multiple arms, 6 studies NS

*RCT* randomized controlled trial, *NS* not stated

The sample size of HL patients varied considerably with 78 % of studies including a sample of 50 or more HL patients. For the 53 cross-sectional studies, the median sample size was 135 (range 15–1843) and for the 12 longitudinal studies the median sample size was 51 (range 12–3208). The majority of study designs were cohort (*n* = 45) and 14 used a comparator cohort design. The most common comparators were the general population (*n* = 23), other cancer patients (*n* = 12) and siblings (*n* = 3). When comparing among other cancer patients, the most common comparator included other types of lymphoma (*n* = 7). The majority of studies relied on remote data collection (*n* = 42), when specified either by mail (*n* = 35) or telephone (*n* = 6), with the remainder conducted in person. The study funding was primarily investigator initiated (*n* = 58) or via a cooperative group (*n* = 7). We did not identify any industry-funded studies. Half (52 %) of the identified studies were published after 2005. Table 2 contains the data extraction results.

**Table 2:** Studies assessing HRQL in Hodgkin lymphoma patients (*N* = 65)

Primary Author	Year	Design	Study Type	Timepoints (as reported)	N (HL Specific)	Age (HL specific)	Tx Status	Measures	Domains Assessed	Quality Score
Fobair P [26]	1986	Cohort	Cross-sectional	Median 9 years post treatment	403	36.3 mean	Off	CES-D, Study-specific questionnaire	Domain, Symptom	6
Kornblith AB [22]	1990	Cohort	Cross-sectional	6.3 years mean post treatment	273	37 mean	Off	PAIS-SR, BSI, POMS, IES, Global Sexual Satisfaction Index	Domain	5
Chao NJ [37]	1992	Cohort	Longitudinal	<1 year post treatment	24	N/S	Off	Study-specific questionnaire	MultiD	2
Kornblith AB [23]	1992	Cohort	Cross-sectional	Mean 6.3 years post treatment	273	37 mean	Off	PAIS-SR, BSI, POMS, Global Sexual Satisfaction Index	Domain	6
Kornblith [48] AB	1992	Cohort	Cross-sectional	Mean 2.2 years post treatment	93	35 median	Off	PAIS-SR, BSI, POMS, IES	Domain	4
Bloom JR [49]	1993	Comparator: Cohort	Cross-sectional	1 or more years post treatment	85	32.3 mean	Off	POMS, CES-D, Social Activity Scale	Domain	6
van Tulder MW [30]	1994	Comparator: Cohort	Cross-sectional	Mean 14 years since diagnosis	81	43.6 mean	Off	SF-36, Maudsley Martital Questionnaire	MultiD, Other	6
Joly F [38]	1996	Comparator: Case Control	Cross-sectional	Mean 10 years since diagnosis	93	42 mean	Off	EORTC QLQ-C30, Study-specific questionnaire	MultiD, Other	6
Norum J [50]	1996	Cohort	Cross-sectional	Median 48 months since diagnosis	42	38 median	Off	IES, VAS	Domain, Other	4
Norum J [51]	1996	Cohort	Cross-sectional	Median 48 months since diagnosis	42	38 median	Off	EuroQol	MultiD	4
Norum J [52]	1996	Cohort	Cross-sectional	Median 48 months since diagnosis	42	38 median	Off	EORTC QLQ-C30	MultiD	3
Abrahamsen AF [29]	1998	Cohort	Cross-sectional	Mean 12 years since diagnosis	459	44 median	Off	Study-specific questionnaire	Domain	6
Kornblith AB [24]	1998	Comparator: Cohort	Cross-sectional	Mean 5.9 years post treatment	273	37 median	Off	PAIS-SR, BSI, POMS, IES	Domain	6
Greil R [13]	1999	Cohort	Cross-sectional	Mean 10.5 years since diagnosis	126	36.9 mean	Off	EORTC QLQ-C30	MultiD	6
Kaasa S [53]	1999	Comparator: Cohort	Cross-sectional	3–20 years post treatment	459	44 mean	Off	SF-36, FQ	MultiD, Symptoms	5
Loge JH [54]	1999	Cohort	Cross-sectional	Mean 12.2 years since diagnosis	459	44 mean	Off	HADS, FQ	Domain, Symptom	6
Loge JH [55]	1999	Cohort	Cross-sectional	Mean 12.2 years since diagnosis	459	44 mean	Off	SF-36	MultiD	6
Van Schaik CS [56]	1999	Cohort	Cross-sectional	Median 21.9 years since diagnosis	33	21.9 median	Off	HUI2, HUI3	MultiD	3
Loge JH [32]	2000	Cohort	Cross-sectional	3–23 years post treatment	421	19–74 years	Off	FQ, HADS, Study-specific questionnaire	Domain. Symptom	6
Barr RD [57]	2001	Comparator: Cohort	Cross-sectional	Completed treatment at least 2 years prior	19	N/S	Off	HUI	MultiD	2
Cameron CL [58]	2001	Cohort	Cross-sectional	Median 7 years since diagnosis	272	37 mean	Off	PAIS-SR, Symptom Report	Domain, Symptom	4
Knobel H [35]	2001	Cohort	Cross-sectional	Mean 9 years since diagnosis	92	37 mean	Off	FQ	Symptom	6
Zabora J [59]	2001	Cohort	Cross-sectional	58 % diagnosed within 90 days from study	135	N/S	Both	BSI	Domain	3
Zebrack BJ [43]	2002	Comparator: Cohort	Cross-sectional	Mean 16.2 years since diagnosis	1843	30.8 mean	Off	BSI	Domain	5
Ganz PA [10]	2003	Comparator: RCT	Longitudinal	Trial registration (since diagnosis)	244	31.4 STLI, 33.7 CMT median	Both	SF-36, CARES-SF, SDS	MultiD, Symptom	4
Gil-Fernández JJ [27]	2003	Cohort	Cross-sectional	Median 7.6 years since diagnosis	46	43 mean	Off	EORTC QLQ-C30, HADS	MultiD, Other	5
Oldervoll LM [36]	2003	Cohort	Longitudinal	Mean post treatment: 6.6 years fatigued patients; 4.9 years non-fatigued patients	53	41 fatigue, 40 non-fatigued mean	Off	SF-36, FQ	MultiD, Symptom	5
Rüffer JU [33]	2003	Comparator: Case Control	Cross-sectional	Median 5.2 years since diagnosis	818	31 median	Off	EORTC QLQ-C30, MFI, Life Situation Questionnaire	MultiD, Symptom	5
Wettergren L [60]	2003	Cohort	Cross-sectional	Mean 14 years since diagnosis	121	47 mean	Off	SEIQol-DW	MultiD	5
Adams MJ [16]	2004	Cohort	Cross-sectional	Median 14.3 years since diagnosis	48	31.9 median	Off	SF-36, Study-specific questionnaire	MultiD	4
Wettergren L [28]	2004	Cohort	Cross-sectional	Mean 13 years since diagnosis	121	47 median	Off	SF-12, SEIQoL-DW, HADS	MultiD, Domain	6
Hjermstad MJ [61]	2005	Cohort	Cross-sectional	Median 16.3 years since diagnosis	496	46 median	Off	FQ	Symptom	6
Ng AK [34]	2005	Comparator: Cohort	Cross-sectional	Median 15 years since diagnosis	511	44 median	Off	FACIT-Fatigue	Symptom	6
Wettergren L [62]	2005	Cohort	Cross-sectional	Median 14 years since diagnosis	121	NS	Off	SF-12, SEIQoL-DW	MultiD	2
Hjermstad MJ [63]	2006	Cohort	Cross-sectional	Median 16.3 years since diagnosis	479	46 mean	Off	SF-36, FQ	MultiD, Symptom	5
Mols F [21]	2006	Cohort	Cross-sectional	5–15 years since diagnosis	132	NS	Off	SF-36, QoL-CS	MultiD	6
Absolom K [64]	2007	Cohort	Cross-sectional	15.7 years mean since diagnosis	50	39.7 mean	Off	SF-12, HADS	MultiD, Domain	5
Aksnes LH [65]	2007	Comparator: Cohort	Cross-sectional	Mean since diagnosis: 14 years for males, 11 years for females	89	35 mean	Off	SF-36, HADS, FQ	MultiD, Domain, Symptom	4
Oldervoll LM [66]	2007	Cohort	Cross-sectional	Mean 17 years since diagnosis	476	46 mean	Off	FQ	Symptom	5
Goodman KA [19]	2008	Cohort	Cross-sectional	Median 12 years post treatment	60	43 median	Off	EORTC QLQ-C30	MultiD	6
Mulrooney DA [67]	2008	Comparator: Cohort	Cross-sectional	At least 15 years since diagnosis	995	NS	Off	FACIT-Fatigue, Pittsburgh Slee Quality Index, Epworth Sleepiness Scale	Symptoms	4
Shimoda S [68]	2008	Cohort	Cross-sectional	Mean 16.5 years since diagnosis	15	NS	Off	HUI3	MultiD	2
Kiserud CE [14]	2009	Cohort	Cross-sectional	Mean 15.2 follow up years	138	45.7 mean	Off	BSFI, Fatigue questionnaire, HADS, SF-36	MultiD, Symptom, Domain	6
Heutte N [11]	2009	Comparator: RCT	Longitudinal	Mean 7.5 years since diagnosis	935	31 median	Off	EORTC QLQ-C30, MFI, Sexual Function Scale	MultiD, Symptoms	6
Brandt J [69]	2010	Cohort	Cross-sectional	Median since diagnosis: HDCT 11 years, conventional chemotherapy 3.5 years	98	46 HDCT, 41 conventional median	Off	EORTC QLQ-C30, EQ-5D	MultiD	6
Klaassen RJ [40]	2010	Cohort	Longitudinal	2 weeks after first course of chemotherapy	49	14.7 mean	Both	PedsQL, HUI2, HUI3, EuroQol, Lanksy Play-Performance Score	MultiD, Other	4
Klaassen RJ [41]	2010	Comparator: Cohort	Longitudinal	2 weeks after first course of chemotherapy	49	14.7 mean	Both	PedsQL, HUI2, HUI3, EuroQol, Lanksy Play-Performance Score	MultiD, Other	2
Miltényi Z [17]	2010	Cohort	Cross-sectional	Mean 9.8 years since diagnosis	168	43.11 median	Off	EORTC QLQ-C30	MultiD	6
Baptista RLR [70]	2012	Cohort	Cross-sectional	Median 7 years since diagnosis	200	29 median	Off	MFI	Symptom	4
Khimani N [18]	2013	Cohort	Longitudinal	Median 24 years since diagnosis	273	52 median	Off	SF-36, FACIT-Fatigue	MultiD, Symptom	5
Minn AY [20]	2012	Cohort	Cross-sectional	Median 10.2 years post treatment	71	26 median	Off	EORTC QLQ-C30	MultiD	5
Behringer K [12]	2013	Comparator: RCT	Longitudinal	At diagnosis	3208	36.4 mean	Both	EORTC QLQ-C30, MFI, Sexual Health Scale	MultiD, Symptoms	6
Greaves P [39]	2014	Comparator: Cohort	Cross-sectional	Mean 20.3 years since diagnosis	280	53.1 mean	Off	FACT-BMT, IOC, Study-specific questionnaire	MultiD, Other	5
Hamre H [44]	2013	Comparator: Cohort	Cross-sectional	Median 21.5 years since diagnosis	68	35 median	Off	FQ	Symptom	5
Kim S [71]	2014	Cohort	Cross-sectional	Mean 6.7 years since diagnosis	58	43.3 mean	Off	EORTC QLQ-C30, HADS	MultiD, Domain	6
Roper K [72]	2013	Cohort	Longitudinal	At completion of planned therapy	40	30.9 mean	Off	SF-12, HADS, SDS, IOC	MultiD, Domain, Other	4
Soares A [45]	2013	Cohort	Cross-sectional	Median 7 years since diagnosis	200	29 median	Off	SF-12, QOL-CS, MOS-SSS, MFI	MultiD, Symptom	4
Vissers PAJ [15]	2013	Cohort	Cross-sectional	Mean 5 years since diagnosis	150	47 mean	Off	EORTC QLQ-C30	MultiD	6
Calaminus G [46]	2014	Cohort	Cross-sectional	Mean 15.3 years since diagnosis	725	28.44 mean	Off	EORTC QLQ-C30	MultiD	6
Daniels LA [31]	2014	Cohort	Cross-sectional	Mean 4.6 years since diagnosis	180	46 mean	Off	EORTC QLQ-C30, Fatigue Assessment Scale, Study-specific questionnaire	MultiD, Symtom, Domain	6
Daniels LA [73]	2014	Cohort	Longitudinal	Mean 21 years since diagnosis	43	47 mean	Off	EORTC QLQ-C30, Fatigue Assessment Scale, HADS	MultiD, Domain, Symptom	4
Oerlemans S [25]	2014	Comparator: Case Control	Longitudinal	Mean 4.7 years since diagnosis	180	46.1 mean	Off	EORTC QLQ-C30, HADS	MultiD, Domain	6
Vermaete N [47]	2014	Comparator: Cohort	Longitudinal	Before the start of chemotherapy	12	47 mean	Both	EORTC QLQ-C30, Distress Barometer	MultiD, Domain	5
Kiserud CE [74]	2015	Cohort	Cross-sectional	Median 16 years since diagnosis	131	46 median	Off	Fatigue questionnaire, HADS, SF36,	Symptom, MultiD	5
Husson O [42]	2015	Comparator: Cohort	Cross-sectional	Mean 5.3 years since diagnosis	150	46.6 mean	Off	Fatigue assessment scale	Symptom	4

*Abbreviations*: *Tx* treatment, *MultiD* multidimensional, *HL* Hodgkin’s Lymphoma, *RCT* randomized controlled trial, *EORTC QLQ-C30* European Organisation for Research and Treatment of Cancer Quality of Life Questionnaire, *SF-36/SF-12* Short Form, *HUI* Health Utilities Index, *QOL-CS* Quality of Life-Cancer Survivors, *CES-D* Center for Epidemiologic Studies Depression Scale, *GSSI* Global Sexual Satisfaction Index, *IES* Impact of Event Scale, *VAS* Visual Analogue Scale, *CARES-SF* Cancer Rehabilitation Evaluation System-Short Form, *SDS* Symptom Distress Scale, *SEIQol-DW* Schedule for the Evaluation of the Individual Quality of Life-Direct Weighting, *PedsQL* Pediatric Quality of Life Inventory, *MOS-SSS* Medical Outcomes Study-Social Support Survey, *FACT-BMT* Functional Assessment of Cancer Therapy—Bone Marrow Transplant, *FQ* Fatigue Questionnaire, *MFI* Multi-Dimensional Fatigue Inventory, *FACIT-Fatigue* Functional Assessment of Chronic Illness Therapy-Fatigue, *HADS* Hospital Anxiety and Depression Scale, *BSI* Brief Symptom Inventory, *POMS* Profile of Mood States, *PAIS* Psychosocial Adjustment to Illness Scale, *HDCT* high dose chemotherapy, *STLI* subtotal lymphoid irradiation, *CMT* combined modality treatment, *NS* not stated

### Patient characteristics

Age was reported in 54 studies (83 %). Thirty-three studies reported a mean age of 40 years (SD = 8.5) and 21 studies reported median age of 38 years (range 22–52). In 41 (63 %) studies that reported time since diagnosis, the mean was 11.8 years (*n* = 25) and the median was 12.4 years (*n* = 16). Twelve (19 %) studies reported time post treatment, with a mean of 4.8 years (SD = 1.73) (*n* = 4) and a median of 10.2 years (*n* = 3). Five studies reported only a range of years post treatment (1–23 years).

### Quality of life measures

Studies varied by aspect of HRQL examined as well as by specific instrument used. Table 3 summarizes the various aspects of HRQL by study design (longitudinal vs. cross-sectional). The most commonly used multi-dimensional instruments were the EORTC QLQ-C30 (*n* = 18), the SF-12 or SF-36 (SF-36 Family) (*n* = 18) and Health Utilities Index (HUI) 2, 3 (*n* = 8). Strikingly, all 12 longitudinal studies included a multidimensional scale whereas only 62 % (*n* = 33) of the cross-sectional studies did so. Among cross-sectional studies, the most commonly used measures were the HADS (*n* = 8), Fatigue Questionnaire (*n* = 7), and the EORTC QLQ-C30 (*n* = 7).

**Table 3:** Measure type by study design (*N* = 65)

HRQL measures, *n* (%)^a^	Longitudinal	Cross-sectional
	*n* = 12 (18)	*n* = 53 (82)
Multidimensional	12 (100)	33 (62)
Domain		
Psychosocial	4 (33)	20 (38)
Sexual functioning	2 (17)	5 (9)
Physical	1 (8)	0 (0)
Symptom		
Fatigue	4 (33)	18 (34)
Pain	0 (0)	1 (2)
Other	2 (4)	4 (8)

^a^Percentages do not sum to 100 because multiple measures can be used in the same study, *HRQL* health-related quality of life

Multi-dimensional HRQL scores varied by treatment type [10, 13, 14], age and sex [11], comorbidities [15] or late effects [16–18]. Both Khimani and Adams found significantly lower quality of life among HD survivors who experienced cardiopulmonary late effects [16, 18]. Goodman and Minn found that HD patients undergoing autologous stem cell transplant reported no difference in global HRQL versus the general population, however these patients did experience a decrease in overall cognitive and social health [19, 20]. Finally, Ganz found that HRQL can improve over the trajectory of treatment into survivorship [10] and Mols supported this finding that HRQL continues to improve through long-term survivorship [21].

Among the single domains explored, psychosocial was the most frequently identified with the psychosocial assessments more common in cross-sectional (38 %) compared to longitudinal (33 %) studies. The most frequently used psychosocial instruments included the Hospital Anxiety and Depression Scale (HADS) (*n* = 13), the Brief Symptom Inventory Scale (BSI) (*n* = 6), the Profile of Mood States (POMS) (*n* = 5) and the Psychosocial Adjustment to Illness Scale (PAIS) (*n* = 5). As studies utilized a variety of scales and measures, the results within this domain were inconsistent and the presence of psychosocial distress varied among reports. Early studies indicated that HL survivors experienced increased psychological distress [22–24], which was later supported by Oerlemans [25]; however, several others studies found no difference in the psychosocial distress of HL survivors when compared to healthy controls [26–28].

The second most commonly assessed domain was sexual health (*n* = 7). When assessing sexual health, researchers used four validated sexual health instruments including the Sexual Health Scale, the Global Sexual Satisfaction Index, the Brief Sexual Function Inventory, and the Sexual Function Scale. Four of the studies indicated that HL survivors report increased SX problems [14, 22, 29, 30]. These SX problems can improve over time [11], but may be more long lasting in higher-stage patients [12].

Several of studies used symptom-specific questionnaires (*n* = 29). Of these symptom assessments, the 76 % majority focused on fatigue (*n* = 22). Other symptom assessments included nausea (*n* = 3), energy level (*n* = 1), pain (*n* = 1) and symptom distress (*n* = 1). The three most commonly used fatigue instruments included the Fatigue Questionnaire (FQ) (*n* = 12), the MFI (*n* = 5), and the Functional Assessment of Chronic Illness Therapy-Fatigue (FACIT-Fatigue) (*n* = 3). Results in this domain were more homogenous with multiple studies indicating that HL survivors are at increased risk for fatigue when compared to healthy controls [10, 31–34]. Studies within this domain found correlations between fatigue and depression ([15, 32] and fatigue and cardiopulmonary late effects [18, 35]. One longitudinal study with a small sample size showed that an exercise intervention can improve fatigue in HL survivors [36].

In our review, 63 (97 %) studies used at least one validated instrument. Two exceptions reported by Abrahamsen [29] and Chao [37], used only non-validated, study-specific questionnaires. Six other studies incorporated a study specific questionnaire in addition to a validated instrument. Of these, three included broad questions including demographic, medical and psychosocial information [26, 38, 39], while the other three created assessment tools specific to their study question pertaining to cardiac health [16], patient screening [31], and psychiatric disorders [32].

### Quality assessment

Our quality assessment was based upon six criteria (Table 4). Nearly 86 % (*n* = 56) of all of the studies included more than two demographic variables and clinical characteristics. The majority (*n* = 53, 82 %) also reported quality of life scores—specifically including a mean score and standard deviation (SD) in table or text form. Of the 11 studies that did not fulfill this criteria, five presented the data graphically [10, 12, 40–42]; three used the data to dichotomize groups (e.g., fatigued vs. non-fatigued population) [36, 43, 44]; two provided means scores but no standard deviation [41, 45]; one provided the percentages of negative impact of sexual function used to compare between groups [39], and one reported the percentage of sexual problems [29]. Most (*n* = 53, 82 %) of the studies compared results of individuals with HL with other groups such as siblings, general population or other individuals with cancer and 78 % of the studies included a sample size greater than 50. For the final criteria, 69 % of the studies included an analysis of HL HRQL scores stratified by HL specific characteristics.

**Table 4:** Quality Assessment of Hodgkin Lymphoma Specific Studies

Quality indicator	Longitudinal (*n* = 12)	Cross-sectional (*n* = 53)	Total (*N* = 65)
Sample >50 (HL Specific)	6 (42)	45 (85)	51 (78)
Description of >=2 Demos (HL Specific)	10 (83)	48 (91)	58 (89)
Description of >=2 Clinical Variables (HL Specific)	11 (92)	45 (85)	56 (86)
HL Specific HRQL Scores (from validated measure)	8 (67)	45 (85)	53 (82)
Analysis of HL HRQL Scores × HL Characteristics	8 (67)	37 (70)	45 (69)
HL Results are compared between groups?	9 (75)	44 (83)	53 (82)

*HL* Hodgkin’s Lymphoma*, HRQL* health-related quality of life

### Key articles

The following section highlights specific studies with a focus on their methodological rigor. The 2013 prospective study by Behringer et al. [12] was embedded within German Hodgkin Study Group HD10-HD12 trials. This study included the largest sample within our review, 3208 patients, and followed these patients from diagnosis through up to 27 months of follow-up care. Patients were randomized to varying protocols of HL chemotherapy and radiation, according to each of the three clinical trials. This study ultimately focused on the sexual functioning (SX) domain; however it also collected multi-dimensional HRQL data. Studies instruments included the European Organization for Research and the Treatment of Cancer (EORTC-QLQ-C30), Multi-dimensional Fatigue Inventory (MFI) and a Sexual Functioning Scale. Researchers found that while SX was reduced at baseline, it improved after therapy and eventually normalized in individuals with early stage disease. Within the HD11 trial, which was a randomized comparison of doxorubicin, bleomycin, vinblastine, and dacarbazine (ABVD) versus bleomycin, etoposide, doxorubicin, cyclophosphamide, vincristine, procarbazine, and prednisone (BEACOPP), a small but significant difference of SX symptoms was detected in favor of ABVD. Long-term SX was related more to baseline SX and patient characteristics than to the intensity of treatment. Of note, older age, advanced stage disease, and female gender had an overall negative influence on SX.

From a quality perspective, the researchers used validated, multi-dimensional assessment tools; however they did not report specific means scores or the standard deviation. Instead, they used the HRQL data as independent variables to predict/explain sexual function and reported it in graph format. This absence of summary scores and variance precludes performing meta-analysis from these published data.

In 2003 Ganz et al. [10] conducted the only RCT of HRQL in HL in the US. This study was embedded in a RCT in cooperative Southwest Oncology Group 9133 trial. It included 247 patients in a prospective, longitudinal design, evaluating multidimensional HRQL at baseline, 6 months, 1 and 2 years. Researchers found a statistical difference in HRQL between the two treatments subtotal lymphoid irradiation with combined modality treatment (CMT). Specifically, the CMT arm experienced greater symptom distress, fatigue and poorer overall HRQL; however, by years 1 to 2, patients with in the two groups did not differ in outcomes. The authors also reported that both groups experienced significantly increased fatigue at 6 months from baseline (when compared to healthy populations) and this persisted even 1–2 years after treatment.

This study used a high quality methodology with a prospective, longitudinal, RCT design, a large sample size, and validated multi-dimensional assessment tools; however, like Behringer [12], they did not report specific means scores or the standard deviation, but instead presented trends over time graphically.

Heutte et al. [11] conducted a prospective, longitudinal study assessing HRQL among patients treated on the H8 study in Europe. This study included 935 patients, who were assessed at the end of therapy and serially up 10 years following treatment using the EORTC-QLQ-C30, MFI, and sexual function scale. Their results indicated that the emotional functioning scores were more affected than the physical functioning scores, and that women reported lower HRQL and increased symptom distress than men. All of the HRQL domains they evaluated showed improvement within 18 months of treatment completion with the exception of cognitive function and motivation. The authors suggest that neither of these domains were affected by the HL or treatment. Finally, they found that high levels of fatigue at end of treatment predicted persistent fatigue into long-terma follow-up. They did not find any differences between the treatment groups. Methodologically, this study included a large xsample size, randomization between treatment groups and used validated, multi-dimensional assessment tools. Methodologic concerns included the lack of baseline data (prior to initiation of treatment) and no data obtained during treatment.

Calaminus et al. [46] conducted a cross-sectional study assessing HRQL in pediatric survivors, who were previously treated for HL in German-Austrian RCT studies from 1978 to 2002. This study enrolled 725 participants, who were assessed using the EORTC QLQ-C30. Results were compared to a similarly matched general population sample. Several of the results of this study are similar to Heutte’s: survivors experienced worse emotional and social functioning compared to normal population; females experience lower over-all functioning and higher symptom burden than men; survivors experienced greater symptoms of fatigue and sleep problems. Again, there was no relationship between the treatment types. Methodologically, this study included a large sample size, validated instruments and comparisons between groups. Although it did not follow patients over time, the study does illustrate how a well-designed cross sectional study can support longitudinal findings.

Klaassen et al. [40] examined the ability to detect change over time in four different HRQL measures. In their prospective study of 48 patients, patients were assessed at four points in time: 2 weeks after first course of chemotherapy, during the second course of chemotherapy, during their week of radiation and 1 year after diagnosis. All of the HRQL measures demonstrated significant change from Time 1 to Time 4. In a second study, Klaassen [41] also examined proxy reporting by parents and nurses of children with HL to determine if correlation with children’s report. In this study, the authors found that over the course of treatment there was statistical significant agreement among the child, parent and nurse, as measured by the Spearman rank-order correlation coefficient. Methodologically, this study used validated tools and compared trends over time; however, concerns include its small sample size, no baseline data, no randomization according to the treatment arm or disease stage and the absence of means scores with standard deviations.

Vermaete et al. [47] conducted a longitudinal study to assess fatigue, physical activity and physical fitness in individuals with lymphoma before, during and after treatment. This study included 29 patients with either HL (*n* = 12) or Non-Hodgkin Lymphoma (*n* = 17). Over course of treatment, researchers detected a decline in hemoglobin, physical force and oxygen uptake, and patients reported significantly increased fatigue. This study looks deeper into the complex relationship between fatigue, physical inactivity and deconditioning and support further work in developing exercise interventions. Methodologically, this study used validated tools, obtained baseline prior to the start of treatment and provided mean scores with standard deviation; however, it enrolled a small sample size and did not randomize according to the treatment arm or disease stage.

## Discussion

In our review the majority of studies employed cross-sectional versus longitudinal design and of these cross-sectional studies, 98 % enrolled participants off treatment. These cross sectional studies capture patients within a wide range of time periods after diagnosis, on average, 10 years after treatment. While cross sectional studies provide a “snap shot in time,” analyzing a group one decade after treatment introduces the risk of many confounding variables, which makes it difficult to build a cohesive narrative around the impact of HL and its treatment on HRQL. In terms of assessing HRQL, all longitudinal studies used a multi-dimensional measure compared to 62 % of cross-sectional studies, which focused more on specific domains and symptoms, especially fatigue.

The objective of this review was to systematically identify published studies reporting HRQL in individuals with HL, and to examine the quality of these studies. Our analysis points to several positive trends in the literature. First, it is clear that that over time there has been a growing interest in this topic, as the number of studies that examine HRQL has increased. As noted, half of the studies identified in this review were published after 2005 (*n* = 34). The second positive trend is movement toward more longitudinal studies with nine of the 12 published since 2009. In regard to patient reported outcomes in HRQL, the majority of studies used at least one multidimensional instrument (*n* = 45) and 97 % of studies included at least one validated instrument.

We also noted several concerns with quality of the current literature. The most rigorous methodology employs a longitudinal design with comparison between groups and changes within subjects. Our review identified only 12 longitudinal studies with ten unique data sets. Only five out of the 12 studies followed patients from diagnosis through to post-treatment and of these only two included at least 50 patients. Reporting HRQL data also varied across the longitudinal studies with four reporting results graphically without mean summary scores and measures of variability [10, 12, 40, 41], which prevents further meta-analysis.

Behringer and colleagues [12] illustrate the complex and informative data that can be derived from prospective, longitudinal designs. It provides clinician’s with a deeper understanding of the trajectory of SX from diagnosis through treatment; additionally, it describes how SX differs across treatment groups and identifies those specific populations, who are most vulnerable. This information can assist clinicians in providing anticipatory guidance and targeted-interventions to the most vulnerable populations.

Results from Ganz and colleagues [10] inform clinicians that symptom burden can vary between treatment groups; however fatigue can persist over time. Clinicians could use this information to guide CMT patients to be more realistic with short-term goals immediately after treatment and to initiate more aggressive symptom management interventions. Second, in regard to fatigue, clinicians could use this information to prepare their patients for fatigue, continue to assess for fatigue during follow-up care and to support further research and interventions in fatigue prevention and management.

### Relevance to research and clinical practice

This systematic review reveals the paucity of information on the HRQL impact of initial diagnosis and treatment on HL patients. The majority of published studies are cross-sectional in design and relatively small in size; only 36 studies have sample sizes >100 (Table 2). This limits the application of study findings due to concerns about generalizability and reproducibility. That said, the few longitudinal studies commencing prior to the initiation of treatment, detailed on page 11, provide such information about HRQL trajectory and how it changes over time and by treatment. These findings offer compelling evidence for the need to replicate these measures in future trials.

This study has several strengths. First, we followed PRISMA guidelines to systematically search the literature to capture the complete and relevant published literature. Second, the quality assessment methods provided a standardized measure by which all articles were evaluated. Last, we highlighted the subset of articles which exemplify best practices to examine HRQL in HL patients, with the goal of building upon these methods and findings in future research.

### Limitations

There are limitations to this study. First, because of the lack of RCTs on this topic, we included observational studies, which are more open to bias. Second, we did not conduct an assessment of publication bias as our aim was to analyze the quality rather than to meta-analyze the findings. Last, most HRQL assessments were completed post-treatment so the longitudinal trajectory of HRQL was not captured. More large-cohort, prospective studies are needed to address this limitation.

## Conclusions

HL is a highly curable disease with standardized treatment paradigms over the last two decades. Although the treatment is well established, a knowledge gap still exists in understanding how this diagnosis and its treatment affect the individual’s HRQL from diagnosis though long-term survivorship. Further, with the exception of an ongoing pediatric cooperative group trial, we found no prospective pediatric studies reporting on HRQL in HL from diagnosis to survivorship. Even in adult studies, we identified a substantial void of HRQL data during the active treatment phase. Finally, we identified no industry-funded studies, although this may change with the emergence of novel therapeutics.

With the growing interest and acknowledgement of the importance of HRQL, we recommend that future research studies employ greater methodological rigor by including prospective, longitudinal randomized designs across both treatment and time. Behringer [12], Heutte et al. [11] and Ganz [10], provide a “gold standard” of research studies that not only examine longitudinal effects within subject changes, but also provide comparisons between different treatment regimens. The information generated by these longitudinal studies helps clinicians target vulnerable populations and provide anticipatory guidance to patients. Such studies will generate further data that clinicians can use to address “real life” HRQL problems that patients face on a day-to-day basis. Further, while research supports that HRQL improves after treatment, continued deficits in some patients within the domains of fatigue, sexual and psychosocial health warrants further study with targeted interventions to mitigate the risk of poorer HRQL. Finally, as the focus on HRQL continues to grow in importance, researchers should consider partnering with industry to examine oncologic treatments within the context of how they will impact the patient’s HRQL.

## Abbreviations

*BSI* brief symptom inventory; *CARES-SF* cancer rehabilitation evaluation system-short form; *CES-D* center for epidemiologic studies depression scale; *CMT* combined modality treatment; *EORTC QLQ-C30* European Organisation for Research and Treatment of Cancer Quality of Life Questionnaire; *FACIT-Fatigue* functional assessment of chronic illness therapy-fatigue; *FACT-BMT* functional assessment of cancer therapy—bone marrow transplant; *FQ* fatigue questionnaire; *GSSI* global sexual satisfaction index; *HADS* Hospital anxiety and depression scale; *HDCT* high dose chemotherapy; *HL* Hodgkin’s lymphoma; *HRQL* health-related quality of life; *HUI* health utilities index; *IES* impact of event scale; *MFI* multi-dimensional fatigue inventory; *MOS-SSS* medical outcomes study-social support survey; *MultiD* multidimensional; *NS* not stated; *PAIS* psychosocial adjustment to illness scale; *PedsQL* pediatric quality of life inventory; *POMS* profile of mood states; *QA* quality assessment; *QOL-CS* quality of life-cancer survivors; *RCT* randomized controlled trial; SD standard deviation; *SDS* symptom distress scale; *SEIQol-DW* schedule for the evaluation of the individual quality of life-direct weighting; *SF-36/SF-12* short form; *STLI* subtotal lymphoid irradiation; *SX* sexual functioning; *Tx* treatment; *VAS* visual analogue scale
